# Coverage theories for metagenomic DNA sequencing based on a generalization of Stevens’ theorem

**DOI:** 10.1007/s00285-012-0586-x

**Published:** 2012-09-11

**Authors:** Michael C. Wendl, Karthik Kota, George M. Weinstock, Makedonka Mitreva

**Affiliations:** 1 The Genome Institute, Washington University, St. Louis, MO 63108 USA; 2 Department of Genetics, Washington University, St. Louis, MO 63108 USA; 3 Department of Mathematics, Washington University, 4444 Forest Park Blvd., Campus Box 8501, St. Louis, MO 63108 USA

**Keywords:** DNA sequencing, Coverage, Microbiome, Metagenomics, 05A10, 60D05, 62K05, 92B99

## Abstract

Metagenomic project design has relied variously upon speculation, semi-empirical and ad hoc heuristic models, and elementary extensions of single-sample Lander–Waterman expectation theory, all of which are demonstrably inadequate. Here, we propose an approach based upon a generalization of Stevens’ Theorem for randomly covering a domain. We extend this result to account for the presence of multiple species, from which are derived useful probabilities for fully recovering a particular target microbe of interest and for average contig length. These show improved specificities compared to older measures and recommend deeper data generation than the levels chosen by some early studies, supporting the view that poor assemblies were due at least somewhat to insufficient data. We assess predictions empirically by generating roughly 4.5 Gb of sequence from a twelve member bacterial community, comparing coverage for two particular members, *Selenomonas artemidis* and *Enterococcus faecium*, which are the least ($\sim$3 %) and most ($\sim$12 %) abundant species, respectively. Agreement is reasonable, with differences likely attributable to coverage biases. We show that, in some cases, bias is simple in the sense that a small reduction in read length to simulate less efficient covering brings data and theory into essentially complete accord. Finally, we describe two applications of the theory. One plots coverage probability over the relevant parameter space, constructing essentially a “metagenomic design map” to enable straightforward analysis and design of future projects. The other gives an overview of the data requirements for various types of sequencing milestones, including a desired number of contact reads and contig length, for detection of a rare viral species.

## Introduction

Microbes are both ubiquitous and singularly important to almost every aspect of life as we know it. There is no shortage of remarkable statistics that might be quoted, for example symbiont microbial cells outnumber human somatic cells by about 10 fold in most individuals, microbes represent about half the world’s biomass, and most of the probably more than 10 million bacterial species remain to be discovered. Such numbers contrast starkly with our relatively limited understanding of these organisms, which stems largely from difficulties in isolating and culturing most species in a laboratory setting. However, technology has lately reached the point where comprehensive metagenomic approaches are now being used. Here, whole-genome shotgun (WGS) sequencing is applied directly to the collective DNA of a community of organisms. A number of metagenomes have already been examined in this way (Breitbart et al. 2002; Tyson et al. 2004; Venter et al. 2004; Tringe et al. 2005; Gill et al. 2006; Culley et al. 2006; Angly et al. 2006; Martín et al. 2006; Rusch et al. 2007; Schlüter et al. 2008; Qin et al. 2010; Hess et al. 2011).

Project design remains a significant issue facing metagenomic research. In particular, it is difficult to know how much sequence data should be generated for any particular community. Early projects in the Sanger-era of sequencing often made pragmatic choices based simply on speculation (Handelsman et al. 1998) or budgetary constraints (Kunin et al. 2008). Sequencing was relatively expensive, limiting the amount of data. This meant that while simple metagenomic communities could still be mostly reconstructed (Tyson et al. 2004; Culley et al. 2006), large tracts within highly complex communities would necessarily be left uncharted (Venter et al. 2004; Tringe et al. 2005).

The commonality across all sequencing scenarios is that project success depends strongly on the notion of *covering* (Wendl and Wilson 2008, 2009a, b), i.e. the process that randomly places one-dimensional DNA segments onto larger genomic DNA targets. Venter et al. (2004) summarize the coverage idiosyncrasies of metagenomic sequencing in terms of the differences in both genome size among the member species and among their relative abundances. In essence, if abundance levels are roughly uniform, any single sequencing read is more likely to have come from a large genome rather than a small one. If instead genome sizes are all similar, this read probably represents an abundant species of individuals rather than a rare one. The sampling dynamics of an actual metagenomic project are a community-specific mixture of these two phenomena and the obvious danger is one of missing the proverbial “needle in the haystack” (Kowalchuk et al. 2007). That is, data may not adequately capture a member that plays some particularly vital internal role within the community and/or has some otherwise important biomedical relevance outside the community. The serendipitous discovery of the proteorhodopsins is a good example (Béjà et al. 2000).

**Fig. 1:**
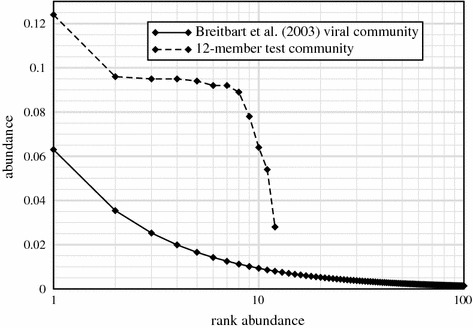
Rank abundance curves are shown for the 12-member test microbial community used here for comparison and for the viral community analyzed by Breitbart et al. (2003). The latter was estimated to have around 1,200 species and to be distributed according to the power law $y = 0.063 x^{-0.831}$, assuming a 50 kb average genome size

Abundance biases are especially important in metagenomic projects because they can be quite extreme. Consider the viral community studied by Breitbart et al. (2003), which was estimated to contain around 1200 species. Its top 10 members, numbering about 0.8 % of those species, account for about 22 % of the community biomass (Fig. 1). Sequence representation will accrue rapidly for them, while their rare counterparts having abundances only on the order of $10^{-4}$ will be much more difficult to recover.

The economics of DNA sequencing have improved dramatically with the commercialization of so-called next-generation technologies (Harismendy et al. 2009), suggesting that comprehensive studies of some of the more complex metagenomes are now becoming feasible. It is likely that the amounts of data that will have to be generated in such projects will be larger than what is now typical. For example, the remarkable figure of 10 Tb (more than 3,000 human genomes) has been floated for a single instance of a soil metagenome (Riesenfeld et al. 2004).

With a few exceptions, the current *de facto* standard methods for making such calculations rely on an elementary extension of traditional single-genome coverage theory (Venter et al. 2004; Tringe et al. 2005; Allen and Banfield 2005; Kunin et al. 2008). Specifically, a species is taken to have a sequence redundancy of $\rho = \alpha \, R\,L / \gamma$, i.e. the respective product of its abundance, total number of project reads and read length, all divided by the species’ sequence-accessible genome size. (Mathematical notation is listed in Table 1). This formula is simply the expected redundancy of data that will represent the species. Substitution into the classical coverage equation (Clarke and Carbon 1976) then yields the expected number of covered bases (Venter et al. 2004; Wooley et al. 2010) as $E\langle C\rangle = \gamma \,(1 - e^{- \rho })$. Lander–Waterman theory (Lander and Waterman 1988) can be further combined to obtain the expected contig length (Rusch et al. 2007) for this species as $E\langle \lambda \rangle = L\,(e^{\rho } - 1) / \rho$. Other results are similarly derived, for example for multiple-read coverage (Chen and Pachter 2005; Tringe et al. 2005).

**Table 1:** Mathematical notation

Variable	Meaning
$\alpha$	Abundance of a species within metagenomic community
$\gamma$	Size in nucleotides of sequenceable genome
$L$	Average length in nucleotides of a sequence read
$R$	Total number of sequenced reads for a community
$\mu$	Expected number of reads for target species: $\mu = \alpha R$
$\varphi$	Probability of a position being covered: often $L/\gamma$
$\rho$	Avg. number of reads spanning a position (redundancy): $\rho = \mu \varphi$
$\eta$	Steven’s series limiter: the smaller of $R$ and $\text{ int}(1/\varphi )$
$B$	Number of sequence gaps in target species (random variable)
$T$	Number of reads hitting target species (random variable)
$\lambda$	Contig size in target species (random variable)
$C$	Coverage: amount of genome covered by reads (random variable)
$V$	Vacancy: complement of coverage (random variable)

While such formulae are attractive because of their simplicity, the salient question is whether they are sufficient for project design. Consider the calculation by Rusch et al. (2007). They predicted that 6-fold Sanger redundancy for a 10 Mb genome at 1 % abundance should give an average contig length of about $E\langle \lambda \rangle = 50$ kb. Now consider another hypothetical species in the same project whose abundance and genome size are only 0.1 % and 1 Mb, respectively, whereby $\rho$ remains at 6-fold. The chance that a randomly-selected read represents this second species has now been reduced by a factor of 100, but the model still predicts $E\langle \lambda \rangle = 50$ kb. Rusch et al. actually reported that most of their data falling outside the dominant species remained “strikingly fragmented”, with the majority not assembling at all. This scenario illustrates a subtle property of expectation-based formulae: measures such as coverage necessarily collapse onto “universal” curves that only depend upon redundancy. In a sense, expectation theory lacks the resolution to say something about specific species, as one might be able to do with a probability model. For instance, the probabilities of the two above species being fully covered are certainly different.

We briefly mention a few other results which, however more sophisticated, are still unsuited to this particular design problem. There is an appreciable body of work in the statistics literature regarding abundance estimation and these methods are readily applied to coverage-type calculations, for example as recently described by Hooper et al. (2009). They propose an expected coverage whose modeling parameters rely on fitting data to a user-chosen kernel function. Reported shortcomings include iterative tuning of parameters, limitations of kernel fidelity, and the need to discard certain portions of the data to preserve the model’s integrity. Perhaps even more important is that calculations can only be made once the project is already underway, having generated enough data for parameter-fitting. The model described by Breitbart et al. (2002) has similar technical issues and does not account for variation in genome size. Alternatively, Wendl (2008) developed the density function for the project-wide number of sequence gaps, but that equation also does not adequately consider the sampling biases mentioned above. Stanhope proposed an approximation model (Stanhope 2010) based on the idealized “occupancy” concept of covering (Wendl 2006b). That approach either takes all species at uniform genome size and abundance, or requires speculative distributions for these unknowns. Finally, there are scattered rules-of-thumb (Dutilh et al. 2009; Riesenfeld et al. 2004) whose origins are not entirely clear and upon which we also comment further below (Sect. 3.1).

These observations collectively point to the need for improved theoretical tools to quantify the metagenomic sequencing process. We propose several such results here. Most are corollaries of a generalization of Stevens’ theorem (Stevens 1939; Fisher 1940; Solomon 1978; Wendl and Waterston 2002), suitably extended to account for the distribution aspect of multiple species and its ensuing “abundance bias”. Like all of the methods above, this work does not strictly consider effects related to particular DNA sequence or instrumentation biases, within-species variation, or choices regarding computational processing. Consequently, we view it merely as another installment within a broader research program of metagenomic sequencing theory.

## Results

The basic premise is to develop useful and rigorous quantitative tools for designing metagenomic projects based on the community members and the level at which one desires to characterize them. The goal might range anywhere from light sampling simply to estimate community membership, to reconstructing the dominant species, to fully recovering an extremely rare member within a very complex constituency. Consequently, we will speak of the *target species* as the basis of design. Species that are more readily accessible to sequencing than the target will almost certainly be even better characterized, while the converse is true for less accessible members. This is an inherent property of all random metagenomic sequencing.

The concept of a “target species” is implicit in expectation models and enables quantitative analysis without having to first speculate closures for the invariably unknown properties of the larger metagenomic community. This aspect is enormously practical. The closure problem is necessarily present for semi-empirical models (Hooper et al. 2009; Breitbart et al. 2002), but our theory does not depend on closure estimates.

### Generalization of Stevens’ theorem

The problem of covering a one-dimensional domain with finite segments had been examined for some time before being solved successfully by W. L. Stevens in 1939 using a form of the well-known probability concept of inclusion–exclusion and a clever geometric observation (Stevens 1939; Fisher 1940; Solomon 1978). We generalize this result to the scenario of covering one particular domain from among a population of distinct domains. The abstraction is clearly applicable to metagenomic sequencing.

Consider a case in which $R$ reads of length $L$ have been processed and define the Bernoulli probability, $\alpha$, as the chance that a randomly selected read represents the target species. This parameter, often understood as the “abundance”, is project-dependent. Also, let $\varphi$ represent the probability that this read covers a particular base position within the target species’ genome. It may simply be $L / \gamma$, or it might be assigned other values to account for overlap detection (Lander and Waterman 1988) and/or the effects of bias (see below). We can now state the following salient result.

#### **Theorem 1**

 (Gap Census) If $B$ is a random variable denoting the number of sequence gaps within the target species’ composite genome, then the probability of $k$ gaps is

$$\begin{aligned} P(B=k) \,=\, {R \atopwithdelims ()k} \sum _{\beta = k}^{\eta } \, {R - k \atopwithdelims ()\beta - k} (-1)^{\beta - k} \, \alpha ^{\beta } \left(1 - \beta \varphi \right)^{\beta - 1} \left(1 - \beta \varphi \alpha \right)^{R- \beta } \end{aligned}$$

for $0 < \varphi < 1$ and $0 < \alpha \le 1$, and where $\eta$ is the smaller of $R$ and $\text{ int}(1/\varphi )$. The latter quantity represents the maximum number of reads that can be placed without overlap on the target and arises from Stevens’ geometric observation (Stevens 1939; Solomon 1978). Stevens’ original theorem is readily shown to be a special case for $\alpha =1$. 

This theorem can be applied either directly, or in various derivative ways to obtain rigorous probabilistic quantifiers for metagenomic sequencing. We discuss two of the more useful implementations in Sect. 2.2: the probability of complete target species coverage and the probability that the average size of contiguous regions of coverage in the target exceeds some threshold. (There are other possibilities, though of lesser practical interest; Roach 1995). Finally, we give another handy formula for community sampling, not related to Theorem 1, but derivable rather from elementary considerations.

### Implementations of Theorem 1 for metagenomic sequencing

As alluded to in the above discussion of expectation models, let $C$ and $\lambda$ be the respective random variables representing the number of base positions covered in the target species’ composite genome and the length of a contiguously covered segment, i.e. a “contig”.

#### **Corollary 1**

 (Complete Coverage) Complete coverage of the target species, $C = \gamma$, also occurs by virtue of all gaps being filled, i.e. $B = 0$. The probability of this event is

$$\begin{aligned} P(B=0) = \sum _{\beta = 0}^{\eta } {R \atopwithdelims ()\beta } (-\alpha )^{\beta } \left(1 - \beta \varphi \right)^{\beta - 1} \left(1 - \beta \varphi \alpha \right)^{R - \beta }. \end{aligned}$$

This is a high standard of coverage. More relaxed conditions based on contig size are also relevant (Roach 1995; Stanhope 2010). Here, we exploit the fact that $C \rightarrow \gamma$ much more rapidly than $E\langle \lambda \rangle \rightarrow \gamma$. That is, coverage increases appreciably faster than contig size, with a large fraction of the process existing in a state of high or even nearly complete coverage, yet still having numerous small gaps (Roach et al. 1995). This phenomenon is nicely illustrated by considering the last few events of the process, where the remaining tiny gaps are closed just before attaining complete coverage. It is only here that $E\langle \lambda \rangle$ grows rapidly as $\dots , \gamma /3, \gamma /2, \gamma$. The effect has been confirmed empirically from the earliest sequencing projects (Fleischmann et al. 1995) and holds for metagenomic projects, as well (Martín et al. 2006).

#### **Corollary 2**

 (Average Contig Size) If coverage is almost complete, the average contig length is, to a very good approximation, a function only of the target size, $\gamma$, and the (random) number of gaps, $B = k$, and is likewise itself then a random variable. The tail probability of a value at least $\gamma / k$ is

$$\begin{aligned} P\left(E\langle \lambda \rangle \ge \frac{\gamma }{k}\right) \,\approx \, \sum _{j = 0}^{k} P(B=j), \end{aligned}$$

where $k \not = 0$ and where the coverage provision can be checked in any suitable way. For example, if $E\langle V \rangle$ is the largest allowable fractional vacancy for the target, say 1 %, then a simple corollary of expectation theory, $\rho \ge \ln (1/E\langle V \rangle )$, might be used. This result can also be generalized by replacing $\gamma$ with $E\langle C\rangle$ (see above), though at an obvious additional degree of approximation.

### Formula for community sampling

Sequencing can also be used in a diagnostic capacity to assess what species are present in a community (Eisen 2007; Kunin et al. 2008). In the simplest case, coverage structure and contiguity are subordinated by raw counts of reads, especially if their lengths are sufficient to identify species merely by alignment against reference sequences.

#### **Theorem 2**

 (Read Count) Let $T$ be the random variable representing the number of reads hitting the target species. Its distribution is Poissonian, $P(T = k) = \mu ^k \exp (-\mu ) / k!$, with a rate $\mu = \alpha R$.

### Numerical evaluation

Theorem 1 and its corollaries have a number of interesting mathematical properties, the most relevant here of which is the convergence rate. Evaluation requires summing terms that are themselves products of progressively larger and smaller numbers. Consequently, round-off error overwhelms slowly converging series unless extended precision arithmetic is employed. While such is required for much of the parameter space, standard precision can be used for Corollary 1 if the heuristic

1 $$\begin{aligned} \varphi \,\ge \, \frac{\ln (\alpha \,R/\zeta _o)}{\alpha \,R} \end{aligned}$$

is satisfied, where $\zeta _o$ is a constant having an empirically determined value on the order of 10. The accessible range is then roughly $P(B=0) > 10^{-3}$, which includes most scenarios of practical interest.

### Parameter estimation

The formulae above can be used either parametrically or applied for specific species. In the former role, calculations will reveal the attributes of the most extreme member, i.e. its size and abundance, that could be captured for a given P-value and amount of data. In the latter, specific estimates of $\varphi$ and $\alpha$ can be used to determine the required data for a given probability or *vice versa*. Estimates for $\varphi$ are straightforward, for example one can take advantage of the fact that bacteria largely fall within $1 \le \gamma \le 5$ Mb if setting $\varphi = L / \gamma$, as discussed above. Conversely, $\alpha$ can be approximated in various ways, including 16S rRNA screening (Liles et al. 2003; Tyson et al. 2004), or methods that utilize light shotgun data, such as protein-coding markers (von Mering et al. 2007), single-copy single-marker complements to 16S, e.g. *rpoB* (Vos et al. 2011), fitting (Hooper et al. 2009), or probabilistic modeling (Xia et al. 2011).

## Discussion

### Coverage probability as a design variable

We already mentioned above some of the shortcomings of using an expectation-based quantity such as $E\langle C\rangle$ as a *measure* for the metagenomic design problem. While Stanhope (2010) is similarly critical, several additional factors support replacement with a probability-based metric, such as $P(B=0)$ in Corollary 1.

The more obvious issues are based on the ensemble nature of expectations themselves. That is, they only characterize trials collectively and not necessarily any single one taken alone. In most instances, variances will not be terribly large compared to respective expectations. For example, the expected number of reads hitting the target species is $\alpha R$ (Theorem 2) with a standard deviation of $\sqrt{\alpha R}$ (Feller 1968) and the deviation in coverage $E\langle C\rangle$ is approximately $\sqrt{\gamma \exp (-\rho )}$ (Wendl 2006a). Consequently, this aspect is the source of *some* uncertainty, but not its main contributor.

The much more substantive concern is actually based on the sensitivity of predictions to small changes in the measure itself. Let us first be clear about the differences in what these measures mean. $E\langle C\rangle$ represents the desired percentage of bases recovered from the target species and its value is typically chosen as something approaching, but not actually equal to 100 %. (That case is mathematically undefined). Conversely, $P(B=0)$ is the actual probability of 100 % coverage and would be picked in roughly the same context as statistical power, e.g. 90 %.

**Fig. 2:**
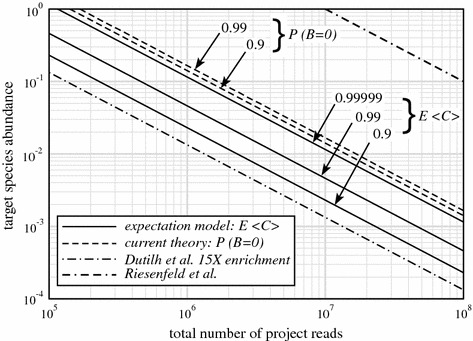
Abundance versus required number of project reads for a 1 Mb target species using 100 bp reads as specified by various theories. Here, $E\langle C\rangle$ is plotted in its fractional context, i.e. as the quotient of covered bases to the genome size. The rule-of-thumb given by Dutilh et al. (2009) is plotted for an enrichment factor of 15. The rule described by Riesenfeld et al. (2004) is plotted for the metagenomic redundancy of 1,000, which leads to their sometime-quoted figure of 10 Tb for a soil metagenome

Figure 2 shows the characteristics of both measures for a 1 Mb target species using 100 bp reads. Here, the expectation results were plotted according to $\alpha R = - \ln (1 - E\langle C\rangle )/\varphi$, which follows directly from the traditional coverage equation. $E\langle C\rangle$ gives an extremely wide range of predictions for the required data. The physical spread of curves is much greater, c.f. the distances between constant-value lines of 0.9 and 0.99 for $P(B=0)$ and $E\langle C\rangle$, which is basically a consequence of the latter’s long asymptotic tail (Wendl and Barbazuk 2005). This is further exacerbated by the somewhat subjective nature of choosing values of the measure itself. Consider that picking $E\langle C\rangle$ “close to 100 %” usually means anything between roughly 99 % (Bouck et al. 1998) and 99.996 % (Green 2001) and these bounds translate to over a two-fold difference in the required data! In short, $E\langle C\rangle$ is inherently ambiguous because of subjective thresholds chosen from within an extremely sensitive sub-domain of this function. Conversely, there is very little ambiguity in using $P(B=0)$; it is chosen within a fairly narrow range for which the lines are very closely spaced.

A final argument, compelling more from an empirical standpoint, is that $E\langle C\rangle$ uniformly specifies fewer required data than $P(B=0)$. Most projects that relied on the former measure reported significant assembly and contiguity problems (Venter et al. 2004; Tringe et al. 2005; Rusch et al. 2007), which seem to be at least partial by-products of having insufficient data. It is also consistent with a more general opinion that current levels of redundancy are inadequate for resolving lower-abundance organisms (Venter et al. 2004; Allen and Banfield 2005; Gill et al. 2006; Rusch et al. 2007; Nicholls 2007; Kunin et al. 2008; Schlüter et al. 2008; Wooley et al. 2010).

Figure 2 also shows two rules-of-thumb gleaned from the literature: the product of target species enrichment and redundancy should be at least 20 (Dutilh et al. 2009) and the metagenome redundancy should be around 1000 (Riesenfeld et al. 2004). The former is plotted for an enrichment factor of 15, again showing clearly insufficient data. This factor is largely arbitrary, being adjustable down to values that move the curve well past those of $E\langle C\rangle$ and $P(B=0)$. Consequently, this rule appears to be entirely too vague and unsupported to be of any practical use. The latter rule is the source of the 10 Tb soil metagenome prediction quoted above and apparently results from the mistaken presumption that the target species redundancy is the product of the species abundance and the redundancy of the metagenome as a whole. As such, it is also unsuitable for further use.

Lastly, we comment on another class of models based on contig length. The standard expectation result, quoted above (Sect. 1), is readily derivable as the ratio of coverage expectation to gap expectation, the latter obtained from Lander–Waterman theory. The formula is often avoided because it is divergent (Lander and Waterman 1988; Roach 1995), a consequence of the fact that gaps approach zero much faster than coverage approaches completion. (This can be demonstrated through simple differentiation.) More recently, Stanhope proposed a metagenomic coverage theory based on the occupancy concept (Stanhope 2010). It furnishes the probability that the largest contig exceeds some length, $f$. Using a property of logarithms (Beyer 1984), the main result in that paper, Eq. 1, can be written in the appreciably simpler form

2 $$\begin{aligned} P(\text{ max} \text{ contig} \ge f) = 1 - \exp \left( -\delta ^f \, \Bigl [(1 - \delta )(2\gamma /L - 3)+2\Bigr ] \right), \end{aligned}$$

where $\delta \approx 1 - [1 - L / (2\gamma )]^R$. This expression is not monotonically increasing, as strict addition of data requires, and violates the boundary condition of 100 % coverage in the limit of infinite $R$. In particular, because $\delta \rightarrow 1$, it is easy to see from Eq. 2 that $P(\text{ max} \text{ contig} \ge f) \rightarrow 1 - \exp (-2) \approx 0.865$.

### Empirical comparison for a 12-member microbial community

On the more pragmatic side, a model’s ability to make worthwhile predictions can be assessed empirically. Here, we compare Corollary 1 to the data obtained from a 12-member bacterial community for which we generated roughly 4.47 Gb of sequence (about 46 million reads) from 1 lane on an Illumina GA-IIx instrument. Table 2 shows the project parameters, where “data” and “size” indicate the total amount of data generated for each genome and actual genome size, respectively. The “depth” column is their quotient, representing the average number of reads spanning each position in the genome, while the “vacant” column indicates the amount of genome remaining uncovered in the assembly. Though having only mild complexity, abundance bias is certainly evident in this population, given that the ratio of highest to lowest abundances exceeds a factor of 4. Larger values are admittedly more common, for example Breitbart et al. (2003) estimate a ratio of more than 300 (Fig. 1). However, the assemblies for such communities generally remain fragmented (Rusch et al. 2007; Hess et al. 2011) and are therefore unworkable as comparisons for the metric $P (B = 0)$. The community in Table 2 is highly redundant, averaging roughly $125\times$ data per species, with the rarest member, *S. artemidis*, still surpassing $50\times$. Consequently, this community is a rigorous, if preliminary test of the theory’s ability to account for abundance bias.

**Table 2:** Sequence data for 12-member microbial community

Species (NCBI accession number)	Depth	Size	Vacant	Data	$\alpha$
	(fold)	(Mb)	(kb)	(Mb)	
*E. faecalis* (AEBQ00000000)	142.5	3.00	2.26	427.4	0.096
*E. coli* (AJGD01000000)	62.8	4.57	2.16	287.1	0.064
*F. prausnitzii* (AECU00000000)	80.7	2.96	3.24	239.0	0.054
*S. artemidis* (AECV01000000)	56.8	2.22	2.19	126.0	0.028
*E. faecalis* (AEBB00000000)	139.2	2.85	1.00	396.6	0.089
*E. faecalis* (AEBP00000000)	115.1	3.01	3.45	346.4	0.078
*E. faecalis* (AEBF00000000)	148.5	2.83	1.64	420.1	0.094
*E. faecalis* (AEBD00000000)	147.7	2.88	1.26	425.5	0.095
*E. faecalis* (AEBN00000000)	132.2	3.12	1.55	412.5	0.092
*E. faecalis* (AEBO00000000)	131.4	3.12	3.36	409.5	0.092
*E. faecalis* (AEBE00000000)	131.3	3.26	2.56	426.5	0.095
*E. faecium* (AEBC00000000)	188.0	2.94	1.25	552.8	0.124

An important, but more subtle aspect in all empirical-theoretical comparisons is controlling for the unavoidable differences that arise as a consequence of project-specific factors, including DNA sequence and instrumentation biases (Harismendy et al. 2009) and the vagaries related to specific combinations of software packages used for processing, alignment, and assembly. In metagenomic projects, we must add inter-strain variation within species as another confounder. These factors, which we will henceforth refer to collectively as “coverage bias”, tend to reduce actual performance below predictions because portions of each species’ genome are inclined against locally spanning reads. While simplistic bias models have been used for posterior fitting (Port et al. 1995; Schbath 1997; Wendl et al. 2001), there is no established, general methodology for resolving this aspect of the design problem *a priori*.

Table 2 shows that the covering process for this community is indeed biased. Specifically, the amount of uncovered genome (vacancy) for each species is on the order of kilobases, despite sequence depths that often substantially exceed $100\times$. Empirical-theoretical comparisons for various other scenarios show that biases do not begin to manifest themselves until significant amounts of coverage have been obtained (Wendl and Barbazuk 2005; Wendl and Wilson 2008). In other words, it is not unusual that much of a genome has little to no bias and closely follows theoretical coverage predictions, some fraction is moderately biased and consequently more difficult to cover, and a small amount is extremely averse to being covered. In essence, the amount of the genome that is accessible to “routine sequencing” is somewhat smaller than the actual genome size (Thousand Genomes Project Consortium 2010; Ajay et al. 2011). This aspect can be particularly problematic for an analysis such as ours, which relies on “100 % coverage” as its metric.

**Fig. 3:**
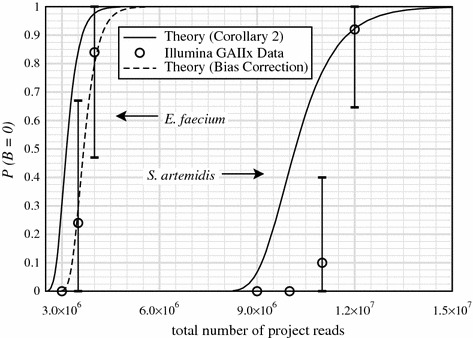
Comparison of data from bacterial community in Table 2 (*circles*) to the probability of total genome coverage given by Corollary 1 (*solid curves*). Each datum represents the average of 50 random drawings from $\sim$46 million reads, where a corresponding indicator variable was set to 1 or 0 depending upon whether total coverage was achieved or not, respectively. *Error bars* are plotted at one standard deviation. *Dashed curve* represents simple bias correction for *E. faecium* in the form of a 3 % lowered relative read length from $\varphi = 3.046 \times 10^{-5}$ to $2.95 \times 10^{-5}$

We compare Corollary 1 specifically to *S. artemidis* and *E. faecium*, which are the least ($\sim$3 %) and most ($\sim$12 %) abundant species in this community, respectively (Fig. 3). Following the above observations, we estimate each species’ accessible genome size as a minimum breadth of coverage obtained after repeated sampling and assembly of a number of reads $R$ such that $P (B = 0)$ is close to unity. For example, *E. faecium* (NCBI accession: AEBC00000000) has an actual genome size of 2,936,981 bp and $R = 4.5\times 10^6$ implies $P (B = 0) \approx 0.997$ for this species, given an average read length of $L = 100$. We then did 50 separate assemblies of 4.5 million reads, all randomly-chosen without replacement, and found each assembly attained coverage of at least 2,933,000 bp. This figure is taken as the amount of the *E. faecium* genome that is routinely accessible to sequencing and the remaining 3,981 bases (0.14 %) are taken to be non-compliant. (Note that roughly 1.25 kb still remains uncovered, even after 188-fold redundancy for this species!) A similar calculation yields an estimate of 2,211,400 accessible bases from the total genome size of 2,215,616 of *S. artemidis*. We deemed 50 simulations per datum to be sufficient, given that the maximum coefficients of variation (quotient of standard deviation and mean) for *E. faecium* and *S. artemidis* were 0.000083 and 0.000185, respectively, for all the assemblies represented in Fig. 3.

The plots show reasonable agreement when considered in light of the bias problem. Although our elementary truncation procedure referenced above corrects somewhat for the worst factors, it unquestionably falls short. If biases are “simple”, meaning relatively benign and not distributed in complicated or extreme ways, it may be possible to further compensate by artificially lowering the read length to simulate less efficient covering. This procedure is demonstrated on *E. faecium*, where we reduced $\varphi$ by about 3 %, from its actual value of $3.046 \times 10^{-5}$ to a compensatory value of $2.95 \times 10^{-5}$, thereby fitting Corollary 1 almost exactly to the data. Conversely, coverage biases can also be stronger and more complicated. In such instances, simple read reduction will not help substantially, as is clear in the case of *S. artemidis*. Broadly speaking, it is difficult to characterize biases *a priori* to a degree that could be formally incorporated into a model. This remains a major unsolved problem in genomic coverage theory.

### Empirical simplification and the metagenomic design map

Theorem 1 is completely general in that it describes probability as a function of all four independent variables: $P = P(B, R, \alpha , \varphi )$. Metagenomic sequencing projects impose additional *empirical* constraints on these variables such that, to a very good approximation, $\alpha$ and $R$ act as a product rather than independently (implied in Fig. 2 for $B = 0$ and demonstrated in Methods), effectively reducing the problem to just three variables for gap census, $P = P(B, \alpha R, \varphi )$, and two for coverage, $P = P(\alpha R, \varphi )$. Contrast this to the functional dependence of $E\langle C\rangle$ on only a single variable, the redundancy, which lumps $\varphi$ into the product $\alpha R \varphi$.

**Fig. 4:**
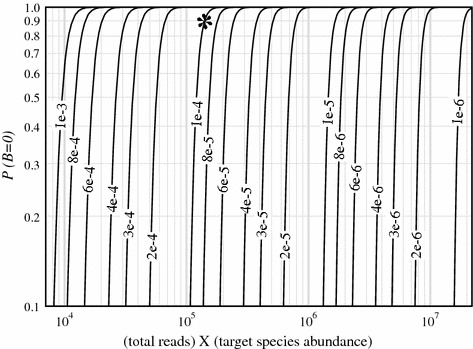
The metagenomic sequencing project design map. Coverage probability is plotted as a function of the product $R \cdot \alpha$ for various values of $\varphi$. Example scenario discussed in Sect. 3.3 is denoted by the *asterisk*. *Curves* do not include any compensation for bias

With respect to coverage, the two-variable dependence enables us to construct what is essentially a “design map” for all metagenomic projects in the form of a single plot (Fig. 4). Assuming estimates of $\varphi$ and $\alpha$ are available for the target species, one simply picks the covering probability on the ordinate, moves horizontally from there to the intersection with the appropriate $\varphi$ curve, then moves vertically down and to the corresponding $\alpha R$ value on the abscissa. The required number of reads for the project is then found by simply dividing this value by $\alpha$. The largely vertical stature of the curves reiterates the observation that predicted data requirements are relatively insensitive to the chosen measure.

Let us illustrate the process with a brief example. Suppose our hypothetical 1 Mb target discussed above in the context of the Rusch et al. (2007) project is to be fully recovered at 90 % power using 100 bp reads. This scenario is denoted by the asterisk in Fig. 4 and corresponds to an abscissa value of roughly $\alpha R = 1.4 \times 10^5$, or a target redundancy of $14\times$. Given its abundance of 0.1 %, the total number of project reads is then about 140 million, or 14 Gb of total sequence data. For comparison, we cite Rusch’s actual figure of about 6.4 million assembled Sanger reads (5.9 Gb of data), as well as the expectation-based prediction of 92 million next-gen 100 bp reads (9.2 Gb of data), assuming we have chosen $E\langle C\rangle = 99.99$ %. Note that this calculation does not include any reduction of $\varphi$ to compensate for bias, as discussed above.

Let us also illustrate the compounding effect of size by now increasing the target to 10 Mb while holding all other parameters constant. Expectation theory simply multiplies everything by 10, according to the rule that the redundancy is constant if we maintain $E\langle C\rangle = 99.99$ %. That is, 920 million reads would now be generated. However, the probability equation accounts for the fact that it is indeed harder to cover a bigger target with constant-size reads. Instead of simply multiplying by 10 to get 1.4 billion reads, the above calculation procedure specifies 1.65 billion reads, or $16.5\times$ target redundancy at 90 % power.

### Assessing community membership

So far, we have concentrated on the special case $P(B=0)$, i.e. full coverage, as the relevant measure, which will be useful primarily for discovery-oriented projects that rely on assembly of previously unknown species. However, there is also increasing interest in application-oriented projects that seek instead to assess community membership, the goal being to accumulate enough sequence to determine whether a known species is present or not (Eisen 2007; Kunin et al. 2008; Stanhope 2010). Because these will rely on alignment more than assembly, read hits and contig lengths are also relevant, suggesting application of Corollary 2 and Theorem 2.

**Fig. 5:**
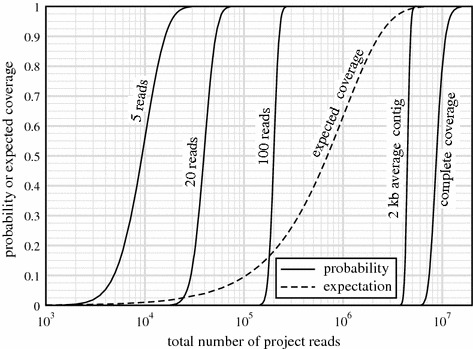
Quantification of various sequencing milestones when using 100 bp reads for a 50 kb target at an abundance of $0.0005$ (i.e. 0.05 %). The curve for expected coverage is computed from classical theory, while probability results are calculated from Corollaries 1 (complete coverage) and 2 (average contig size $\ge$2 kb, implying upper limit of 25 gaps) and Theorem 2 (number of reads hitting target, here $\ge$5, $\ge$20, or $\ge$100, without regard to their associated coverage structures). Curves do not include any compensation for bias

Consider the example of a 50 kb target at 0.05 % abundance (Fig. 5), which is characteristic of a relatively rare virus. Assuming a read length of 100 bp, i.e. $\varphi = 0.002$, we plot a broad spectrum of representative sequencing milestones. (These are not necessarily indicative of the minimum or maximum required amounts of information to reliably indicate species presence, since those undoubtedly vary with the species. The analysis is also predicated on the existence of a well-posed viral reference sequence, which can be an issue in cases of high evolutionary rate). Calculations show that even a moderate number of project reads will very likely result in some number of reads hitting the target organism. For example, for $P (T = k) = 0.95$, the $k = 5$, 20, and 100 thresholds are reached with around $R = 18,\!000$, 55,000, and 235,000 project reads, respectively. These cases are not predicated on any coverage model and it is likely that the reads will exist almost exclusively as singletons without any real contig structure. Expectation theory concurs, for instance it suggests around 20 % coverage for the 100 read hit threshold.

Substantive contigs start to form only at higher levels of coverage. For example, average contig length reaches 2 kb (20 read lengths) at 95 % probability only after about 4.9 million project reads. Expectation theory predicts $>99$ % coverage at this point, consistent with our earlier assertions regarding loss of resolution of $E\langle C\rangle$. In other words, despite “almost” complete coverage, the actual genome is still appreciably fragmented. The apparent contradiction is simply a consequence of the fact that the rate of change of coverage in the late-stages of a project is very small. For comparison, Fig. 5 also shows the complete coverage curve, $P(B=0)$. Here, roughly 11.7 million project reads are required at the same 95 % probability level.

The results for this target virus are readily transformed to other abundance values on the basis that $\alpha$ and $R$ act asymptotically as a product. For example, this same virus at an abundance of 0.5 %, i.e. 10 times more frequent than above, would require approximately 23,500 project reads for 100 hitting reads, 490,000 for 2 kb average contig length, and 1.17 million reads for complete coverage. Note that transforming does not hold in the case of changing read length or genome size for Theorem 1 or its derivative implementations. These expressions would require new evaluation. It does hold for Theorem 2, since that result does not speak to coverage structure and is independent of read and genome length.

Finally, it is interesting to assess the maximum data required for very complex communities. For instance, the (Breitbart et al. 2003) power law estimation suggests the least abundant species in their viral community (Fig. 1) is on the order of $\alpha = 10^{-4}$, about 5 times more rare than the example just discussed. Taking a conservative value of $\varphi = 0.0008$ to account for both bias-related coverage inefficiencies and overlaps in assembling unknown species, Fig. 4 suggests $\alpha \cdot R\approx 20,000$ for high probability of complete coverage. This implies $R = 200$ million reads, or 20 GB of data for 100 bp read length. For equally rare 2 Mb bacteria take $\varphi = 2\times 10^{-5}$, whereby $\alpha \cdot R\approx 1\times 10^6$, indicating 10 billion reads and 1 Tb of data. Such communities are probably near the outer edge of the design space, suggesting an approximate upper bound for the required data.

### Closing remarks

We have described a rigorous mathematical framework for the analysis and design of metagenomic sequencing projects that does not suffer from various resolution, consistency, or closure problems of earlier works. Though it does not address every outstanding issue, including those related to bias, the theory will be useful for a broad spectrum of calculations. We demonstrated several such aspects above, including use of $P(B=0)$ as a coverage metric, empirical comparison to a bacterial community, use of the “product simplification”, and community membership assessment. Numerical implementations of the mathematical results are straightforward, though we are glad to furnish our own code upon request.

Some have argued that sufficiently complex communities will necessarily remain beyond reach (DeLong 2005; Wooley et al. 2010), primarily because of limitations in sampling, while others have maintained that it is simply a matter of generating enough data (Venter et al. 2004; Tyson et al. 2004; Allen and Banfield 2005). This issue may be debatable in the philosophical sense of “proving a negative”. Yet, in a practical sense, our theory furnishes quantitative conditions under which even the most complex metagenomes can be decoded and the least abundant species recovered. Developments in instrumentation continue apace, suggesting many of these communities will be within reach in the near future.

## Methods

### Proof of Theorem 1

Assume all reads are independently and identically distributed (IID) among species in the metagenome, each with a Bernoulli probability $\alpha$ of representing the target species. The probability that $i$ of $R$ reads are indeed derived from the target species is then binomial. Consequently, the probability of $k$ gaps in the target species is obtained by further conditioning Stevens’ theorem (Stevens 1939; Solomon 1978; Wendl and Waterston 2002) upon the number of resident reads $i \in \{k, k+1, \dots , R\}$,

$$\begin{aligned} P(B=k) = \sum _{i = k}^{R} {R \atopwithdelims ()i} \, \alpha ^i \, (1 - \alpha )^{R - i} {i \atopwithdelims ()k} \sum _{j = k}^{\eta } {i - k \atopwithdelims ()j - k} \, (-1)^{j - k} \left(1 - j \varphi \right)^{i - 1}. \end{aligned}$$

Here, $\eta = \text{ min}\Bigl (i, \text{ int}(1/\varphi )\Bigr )$ is the appropriate Stevens limiter. Distribute the binomial and re-order the resulting set of terms, effectively switching the inner and outer summations, to obtain

$$\begin{aligned} P(B=k) = \sum _{j = k}^{\eta } \, \sum _{i = j}^{R} {R \atopwithdelims ()i} {i \atopwithdelims ()k} {i - k \atopwithdelims ()j - k} \alpha ^i (1 - \alpha )^{R - i} (-1)^{j - k} \left(1 - j \varphi \right)^{i - 1}. \end{aligned}$$

The following combinatorial identity can readily be constructed

$$\begin{aligned} {R \atopwithdelims ()i} {i \atopwithdelims ()k} {i - k \atopwithdelims ()j - k} = {R \atopwithdelims ()k} {R - k \atopwithdelims ()j - k} {R - j \atopwithdelims ()R - i} \end{aligned}$$

and substituting this result leads to the factored expression

$$\begin{aligned} P(B=k) = {R \atopwithdelims ()k} \sum _{j = k}^{\eta } {R - k \atopwithdelims ()j - k} \frac{\,(-1)^{j - k} \,f^j\,}{1 - j \varphi } \sum _{i = j}^{R} {R - j \atopwithdelims ()R - i} \, g^{R - i} \, f^{i-j}, \end{aligned}$$

where $f = \alpha \left(1 - j \varphi \right)$ and $g = 1 - \alpha$. Reversing the order of terms for the inner summation and making a suitable change of variables shows that the inner summation collapses via the binomial theorem to $(f + g)^{R - j}$. Theorem 1 follows from straightforward algebra.

### Proof of Theorem 2

Given the IID property of reads, the Bernoulli proposition of either hitting or missing the target species implies binomial distribution. Theorem 2 follows directly from its Poisson approximation (Feller 1968), justified by the fact that $\alpha$ is sufficiently close to zero and $R \gg 1$.

### Derivation of numerical heuristic in Equation 1

The heuristic is based on the notion that rate of growth of successive terms in Corollary 1 is bounded to the degree that the largest one does not overwhelm standard arithmetic precision. The first term is always unity, so we focus on the second. Given $R \gg 1, \alpha < 1$, and $\varphi$ generally less than 0.002, we use asymptotic approximation, finding $R\,\alpha \,\exp \bigl (-\alpha \varphi R\bigr ) \le \zeta _o$, where $\zeta _o$ is our empirically-chosen limiter. Straightforward algebra leads to Eq. 1.

### Collapse of variables

The independent variables in Theorem 1 are governed by $B \ge 0, R > 0, 0 < \!\varphi \! < 1$, and $0 < \alpha \le 1$. However, metagenomic sequencing projects place the further empirical restrictions that $R$ and $\varphi$ are very large and small compared to 1, respectively, and furthermore that $R \gg 1/\varphi$. The last equation means that the overall number of reads in a project is far more than the minimum number required to cover just the target species. These conditions further imply $R \gg \beta$ and $R \gg B$, enabling a significant simplification of the system, wherein the number of independent variables is reduced by one.

The outer and inner combinatorial terms are well-approximated by $R^k / k!$ and $R^{\beta - k} / (\beta - k)!$, respectively, whereby $(\alpha R)^{\beta }$ can be factored. Asymptotic approximation also applies, such that $\left(1 - \beta \varphi \alpha \right)^{R - \beta } \sim \exp (- \alpha R \cdot \beta \cdot \varphi )$. Finally, the series is always limited by $1/\varphi$ rather than $R$, meaning that in all places where $\alpha$ and $R$ appear, they act as a product.

### Sequence generation and analysis

Whole genome shotgun libraries were constructed from 1$\,\upmu$g of starting DNA. The DNA samples were fragmented, end repaired, A-tailed, and ligated. The ligation was size selected for 300–500 bp fragments via ampure beads and 5$\,\upmu$l were then amplified. The final library was quantitated via Qubit and size was verified by Agilent. A 5nM stock was then made from equal pooled volumes of each library followed by qPCR. Sequencing was performed on the Illumina GA-IIx instrument following manufacturer’s instructions. We obtained 50,085,061 reads from the 12 known bacterial genomes, 25,077,278 and 25,007,783 from the two respective ends. A small fraction, 266,146 reads (0.53 %), could not be assigned to any of the 12 species (Table 2).

Analytical processing and assembly of the 12 genomes were managed with the Genome Institute automated pipeline. It initially performs a BWA-style trim (Li and Durbin 2009) to a threshold of q10 on all input instrument data. Reads trimmed to less than 35 bp were discarded. The pipeline then runs Velvet (Zerbino and Birney 2008), which cycles through the 31–35 kmer range, optimizing for the kmer which produces the longest N50 contig length. The entire data set is publicly available through the NCBI Sequence Read Archive (SRA) under the accession numbers listed in Table 2.

BWA (Li and Durbin 2009) was used to align clean paired end reads to the 12 bacterial assemblies, ultimately placing 46,079,563 reads. Up to 5 mismatches were allowed per read, corresponding roughly to minimum 95 % identity. The distribution among the 12 organisms was then assessed using an in-house program called Refcov (Todd Wylie, unpublished) based on the generated alignments. Experimental coverage was then simulated by randomly picking reads from the total pool and assessing subsequent coverage for the target organism again using Refcov. For *E. faecium* (NCBI accession: AEBC00000000), we ran 50 simulations each of 3, 3.5, 4, and 4.5 million reads and for *S. artemidis* (NCBI accession: AECV01000000), which were based on 50 selections each of 9, 10, 11, 12, and 15 million reads. These numbers were based on species abundance within the community in Table 2.
